# Supplementary material for: Advanced Cell Culture Models Illuminate the Interplay between Mammary Tumor Cells and Activated Fibroblasts
Source: Cancers (Basel). 2023 Apr 26;15(9):2498. doi: 10.3390/cancers15092498 (PMC10177476; doi:10.3390/cancers15092498)
Supplement: Supplementary file 1 [file cancers-15-02498-s001.zip › cancers-2283715-supplementary.pdf]

cancers MDPI

Supplementary Materials: Advanced Cell Culture Models Illuminate the Interplay between Mammary Tumor Cells and Activated Fibroblasts

Results

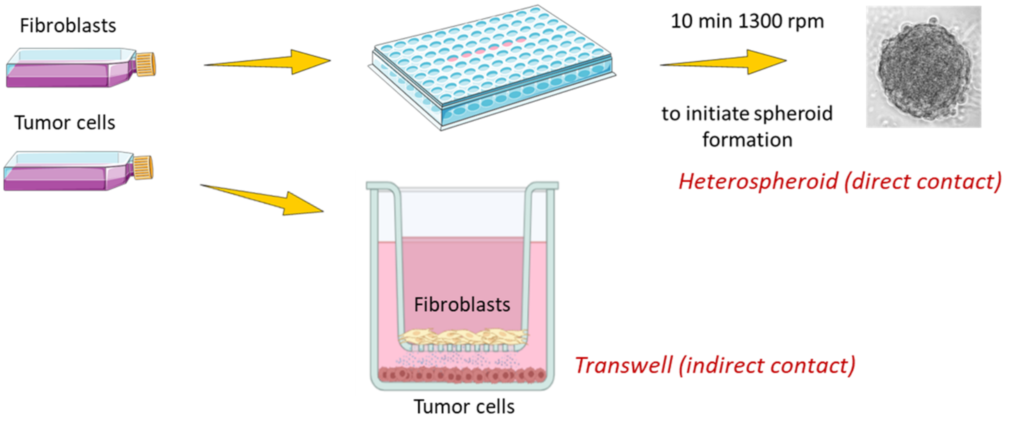

Figure S1. Schematic representation of the two cell culture models developed starting from fibroblasts and tumor cells: co-culture on transwell (indirect contact by paracrine signaling) and heterospheroid (cell-contact-based signaling).

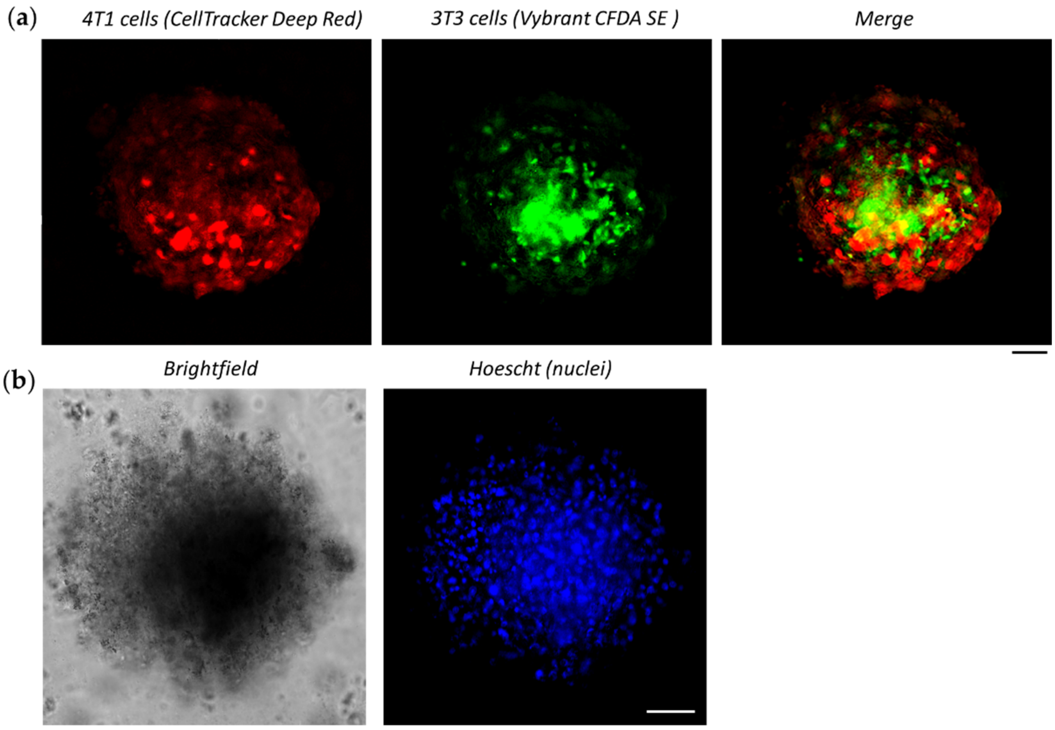

Figure S2. (a) Distribution of 4T1 and NIH-3T3 cells into the heterospheroids. Representative heterospheroid made of 4T1 and NIH-3T3 cells pre-labelled with CellTracker™ Deep Red dye and Vybrant® CFDA SE, respectively (10 x). (b) 4T1 and NIH-3T3 cells into the heterospheroids were labelled with nuclear dye Hoechst 33342 (40 μM) after spheroid formation, to monitor its viability (20 x). Fluorescence images represent a single inner z-stack and were processed in Image J. Scale Bars: 100 μm.

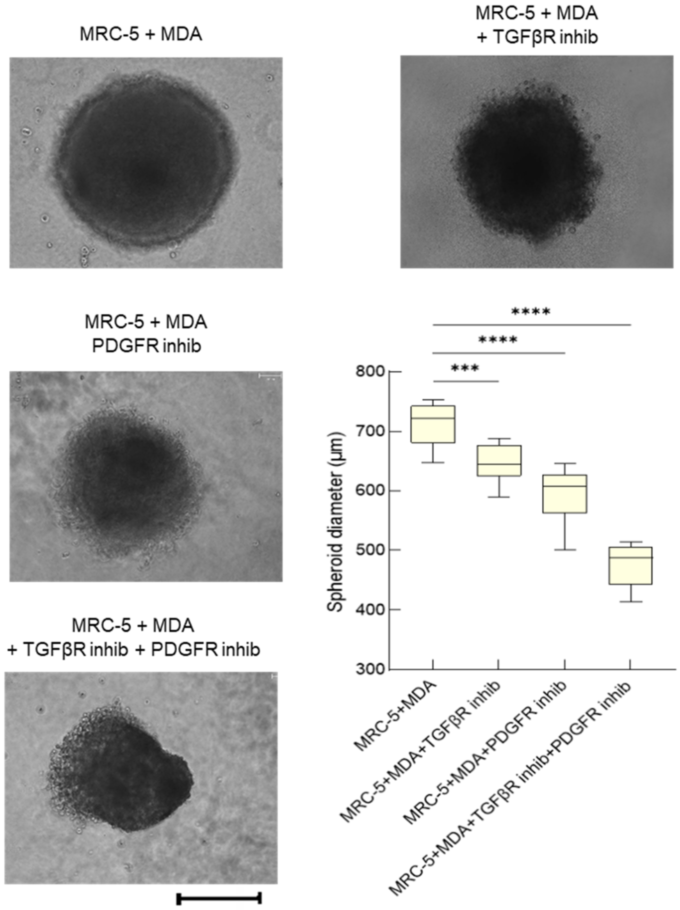

Figure S3. TGF-β and PDGF promote fibroblast proliferation in human heterospheroid cultures. Diameter of 7-days heterospheroids (MRC-5/MDA-MB-231) exposed or not for 7 days to 10 μM TGF-βR inhibitor (SB431542), or 1 μM PDGFRα/β inhibitor (CP-673451) or both inhibitors simultaneously. Diameters have been measured on the optical images of the spheroids. Data, represented as box-and-whisker plots, are compared by One-way ANOVA (****P < 0.0005, ***P < 0.001; *n* = 8). Bar: 500 μm.

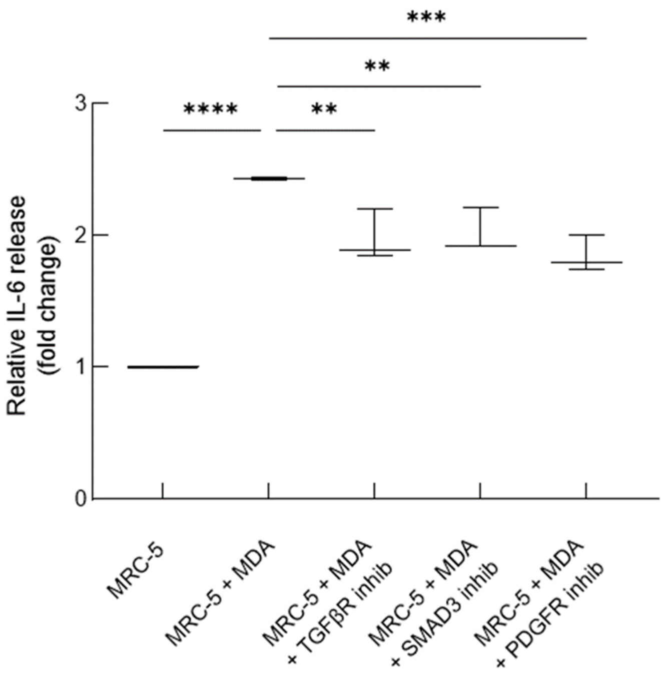

Figure S4. TGF-β and PDGF released by MDA cells promote IL-6 release by activated fibroblasts in heterospheroids MRC-5/MDA. Variation of IL-6 release from the heterospheroids exposed to 10 μM TGF-β receptor inhibitor (SB431542), to 1 μM PDGFRα/β inhibitor (CP-673451) or SMAD3 inhibitor (SIS3). MRC-5 monospheroids have been considered as reference (1). Data, represented as box-and-whisker plots, are compared by One-way ANOVA (****P < 0.0005, ***P < 0.001, **P < 0.01; n = 3)

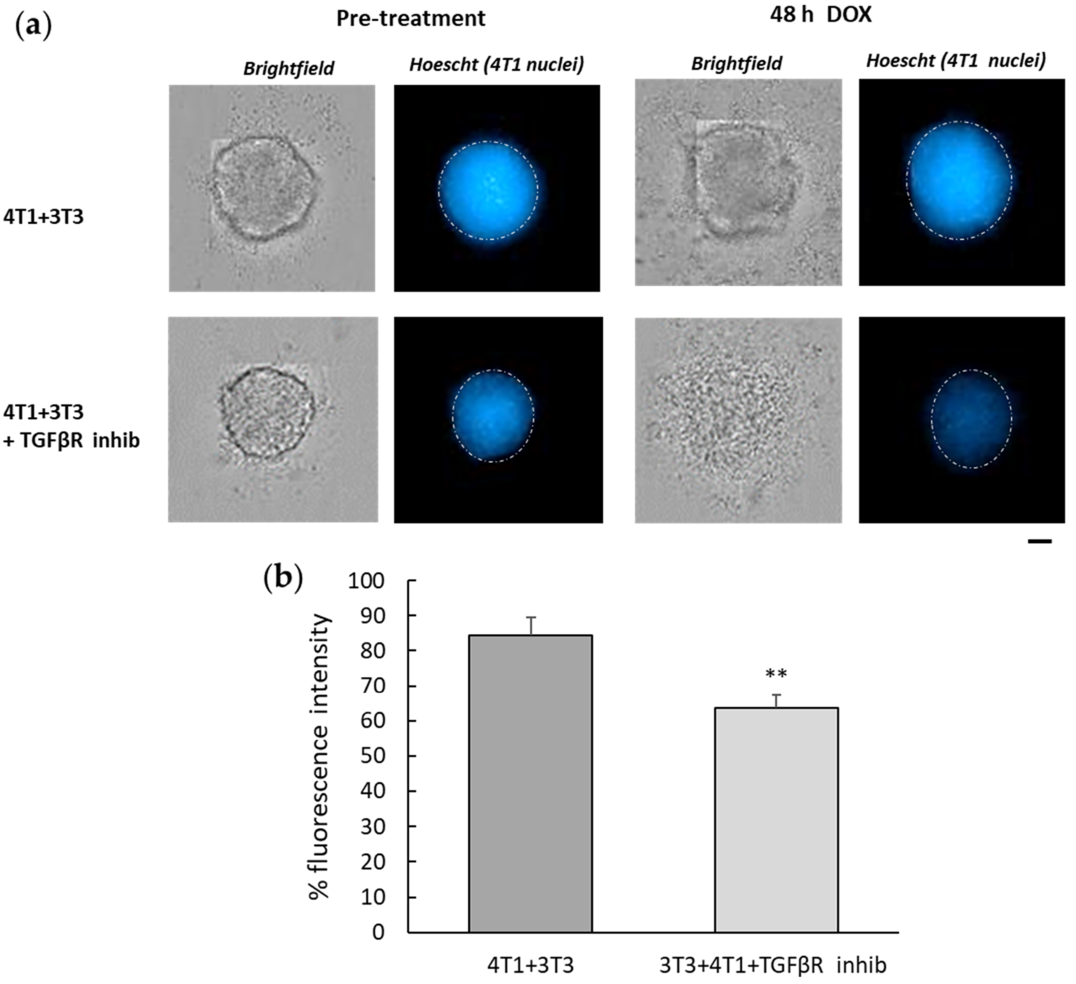

Figure S5. Activated fibroblasts promote 4T1 cell chemoresistance in heterospheroids. (a) Operetta CLS images of heterospheroids grown for 7 days with or without SB431542 (TGFβR inhibitor, 10 μM), prior to and after incubation with DOX (1 μM) for 2 days. 4T1 cells were labelled with nuclear dye Hoechst 33342 (10 μM) before spheroid production. (b) Bar graph shows the Hoechst 33342 signal after DOX treatment divided by the Hoechst 33342 signal prior to treatment expressed as percentage (%e fluorescence intensity). FI has been determined within the white dotted line (ROI). Mean ± SE are displayed (n = 4). ** $P < 0.01$ (Student's t-test). Bar: 200 μm.

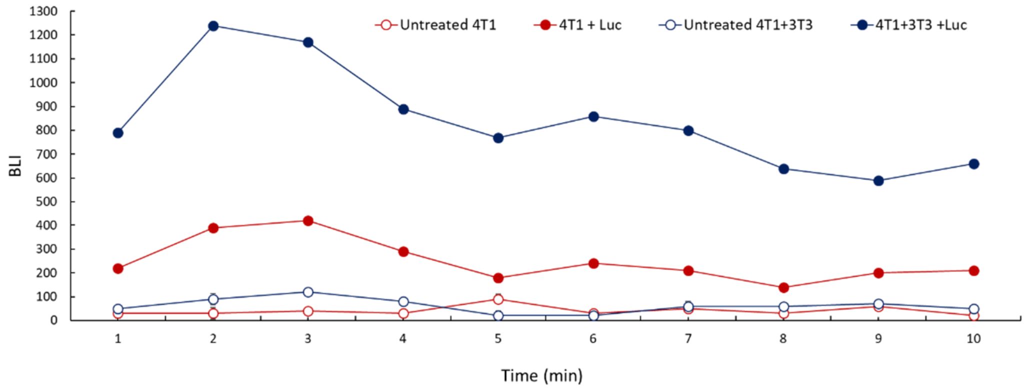

Figure S6. Bioluminescence intensity (BLI) of 4T1 cells increases when they are co-cultured with NIH3T3 in the heterospheroid. BLI of Luciferase+ 4T1 monospheroid or 4T1 in co-culture with NIH3T3 (4T1/3T3 heterospheroids) after exposure over time to 40 µM luciferin. BLI values of untreated mono- and heterospheroids are recorded as reference. Figure is a representative result of different repetitions (at least 3) for each experimental condition.

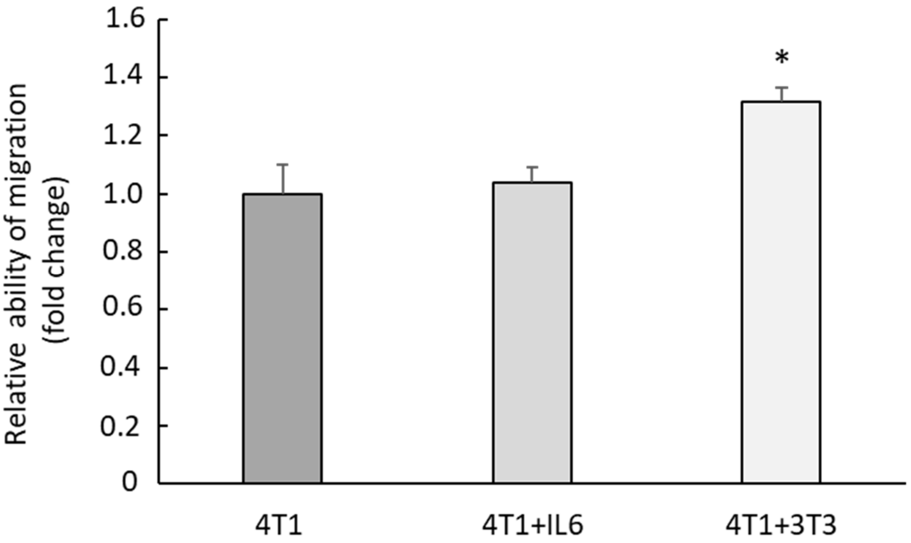

Figure S7. The paracrine effects of activated fibroblasts, but not those of IL-6, stimulate 4T1 cell migration. Bar graph shows changes in the migration ability of 4T1 cells induced by IL-6 (50 ng/mL) or by co-culture with NIH3T3 cells in transwell units. The migration of 4T1 cells alone was taken as a as reference to normalize the data. Mean ± SE (n = 3) are displayed. *P < 0.05 (One-way ANOVA).
